# Significant association between interleukin-10 gene polymorphisms and cervical cancer risk: a meta-analysis

**DOI:** 10.18632/oncotarget.24193

**Published:** 2018-01-12

**Authors:** Chong Guo, Li Wen, Ju-Kun Song, Weng-Jing Zeng, Chao Dan, Yu-Ming Niu, Ming Shen

**Affiliations:** ^1^ Center for Evidence-Based Medicine and Clinical Research, Department of Gynecology and Obstetrics, Taihe Hospital, Hubei University of Medicine, Shiyan 442000, China; ^2^ Department of Dermatology, Suizhou Central Hospital, Hubei University of Medicine, Shiyan 442000, China; ^3^ Department of Oral and Maxillary Surgery, Guizhou Provincial People's Hospital, Guiyang 550002, China; ^4^ Department of Anesthesiology, Taihe Hospital, Hubei University of Medicine, Shiyan 442000, China; ^5^ Department of Urinary Surgery, Taihe Hospital, Hubei University of Medicine, Shiyan 442000, China; ^6^ Center for Evidence-Based Medicine and Clinical Research, Taihe Hospital, Hubei University of Medicine, Shiyan 442000, China; ^7^ Jiangsu Key Laboratory of Oral Diseases, Department of Dental Implant, Affiliated Hospital of Stomatology, Nanjing Medical University, Nanjing 210029, China

**Keywords:** interleukin-10, polymorphism, cervical cancer, meta-analysis

## Abstract

Previous studies have suggested that interleukin-10 (IL-10) polymorphisms may be associated with an increased risk of developing cervical cancer. However, the published results on this subject matter are controversial. The aim of this study was to conduct a meta-analysis of published reports to more precisely investigate the relationship between IL-10 polymorphisms and cervical cancer risk. Five online databases (PubMed, Embase, Web of SCI, CNKI and Wanfang) were searched, and seventeen articles with sufficient quantitative information were included in our meta-analysis. The odds ratios (ORs) and 95% confidence intervals (CIs) were calculated to assess the association between IL-10 polymorphisms and cervical cancer risk. Publication bias, sensitivity and cumulative analyses were also performed to support our findings. Overall, there was a significant association between the IL-10 -1082A > G polymorphism and cervical cancer risk observed in the total population (G vs. A: OR = 1.60, 95% CI = 1.12–2.29, *P =* 0.01, I^2^ = 92.3%; AG vs. AA: OR = 1.34, 95% CI = 1.04-1.74, *P =* 0.03, I^2^ = 65.9%; AG + GG vs. AA: OR = 1.58, 95% CI = 1.11–2.25, *P =* 0.01, I^2^ = 84.4%), and the same results were obtained in the subgroup analysis. Moreover, the IL-10 -819 T > C polymorphism exhibited a significant, protective effect against cervical cancer. In summary, our meta-analysis suggests that IL-10 polymorphisms may play a variety of roles in regard to cervical cancer risk, especially in Asians.

## INTRODUCTION

Cervical cancer is the second most common form of cancer diagnosed in women and the third leading cause of death from cancer. This accounts for approximately 8% of total cancer cases and cancer deaths in women [1]. In 2012, there were an estimated 527,600 new cervical cancer cases and 265,700 deaths from cervical cancer worldwide, mostly affecting developing countries [1]. Cervical cancer affects the cervix and encompasses squamous cell carcinomas (90%), adenocarcinoma (10%), and other subtypes [2, 3]. Currently, a combination of surgery, radiotherapy and chemotherapy is still the most effective form of treatment for cervical cancer [4–6]. However, any form of treatment inevitably implies severe trauma to the patient, as well as an economic burden and mental stress [7–9]. A variety of risk factors, such as chronic inflammation, unhealthy living conditions, and human papillomavirus (HPV) infections have been proven to increase the risk of cervical tumorigenesis [10–13]. However, although numerous epidemiological and molecular biology-related studies have been conducted, the precise effects of these factors on the process of tumorigenesis process are still poorly understood.

In the last decade, numerous studies have suggested that certain cytokines may play critical roles in the processes of inflammatory cell infiltration and malignant cell transformation [14, 15]. Interleukin-10 (IL-10) is a multifunctional cytokine that is mainly secreted by T helper type2 cells (Th2 cells), monocytes/macrophages, keratinocytes and tumor cells as well, as well as human helpertype2 cells (Th1 cells) [16, 17]. IL-10 exhibits complex biological effects, including the capacity to stimulate mast cells maturation and accelerate the proliferation and differentiation of B cells, restrain type 1 immune responses by inhibiting the production of cytokines such as IL-2, IFN-gamma, and other cytokines, decrease the IFN-gamma production by natural killer cells and interfere with macrophage activation [18]. IL-10 exhibits a dual role during cancer development, inducing both cancer-promoting (immunosuppressive) and cancer-inhibiting (anti-angiogenic) effects [19].

Single nucleotide polymorphisms are one of the most common, heritable variations in the human genome, accounting for more than 90% of all variation [20]. The IL-10 gene is located on chromosome 1q31-32, which spans 4.8 kb, and contains 5 exons and 4 introns that encode 178 amino acids [21]. To date, at least 50 polymorphic loci have been reported, such as -2849, -2776, -2769 and -2763 [22]. The three most common SNPs in the IL-10 promoter region that have been reported to significantly influence gene transcription and expression are -1082A > G (rs1800870), -819T > C (rs1800871) and -592C > A (rs1800872) [23]. Some molecular research has shown that these polymorphisms can influence and/or change the susceptibility of individuals to different forms of cancer, such as head and neck cancer, gastric cancer, leukemia, and others [24–26]. In 2001, Stanczuk et al. published the first case-control study examining the effects of the IL-10 -1082A > G polymorphism on cervical cancer risk, and the results suggested that African women with an AG genotype were at an increased risk for cervical cancer [27]. To date, two meta-analyses have been conducted, examining previously published studies to elucidate the association between IL-10 polymorphisms and cervical cancer risk [28, 29]. However, these meta-analyses have not been comprehensive and have not yielded consistent results.

Therefore, we conducted the present meta-analysis to provide a more precise and comprehensive assessment of the relationship between IL-10 polymorphisms and cervical cancer susceptibility. This meta-analysis was performed according to the guidelines of the Preferred Reporting Items for Systematic Reviews and Meta-Analyses (PRISMA) statement [30]. No ethical issues were implicated in this study because our data were based on previously published reports.

## RESULTS

### Study characteristics

**Figure 1:**
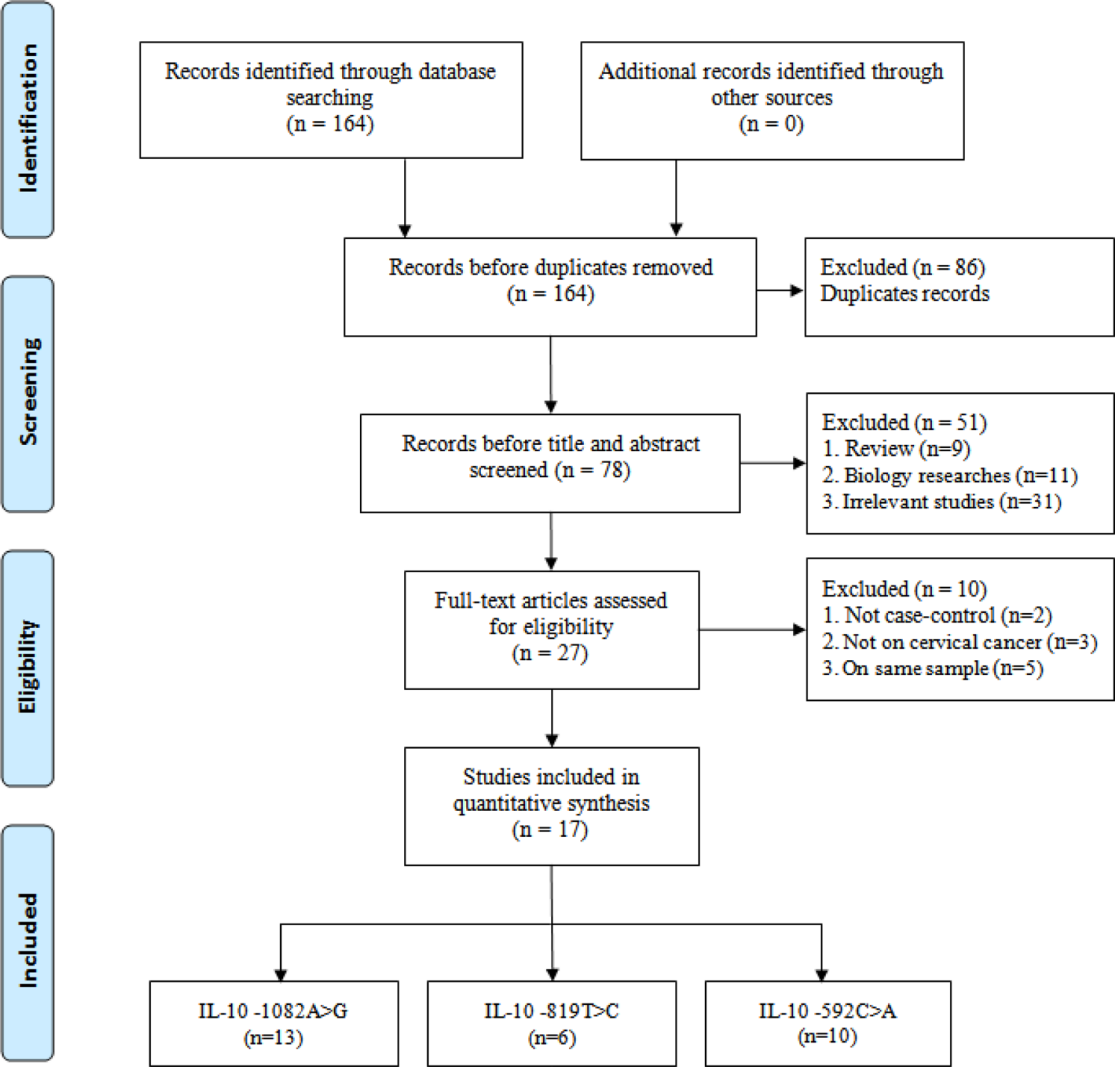
Flow diagram of the study selection process

Initially, 164 relevant articles were identified through our search strategy. The study selection procession is shown in Figure 1. After a comprehensive review, 86 articles were excluded because they were duplicate studies. After screening the title and the abstract, the full text of each article was analyzed. Eventually, 17 published articles (29 studies including polymorphisms at three different loci) involving 4,037 patients and 3,249 controls were included in this meta-analysis [27, 31–46]. Thirteen studies focused on the relationship between the -1082A > G polymorphism and cervical cancer risk [27, 31–42], six studies focused on the relationship between the -819T > C polymorphism and cervical cancer risk [31, 38–40, 42, 43], and ten studies focused on the relationship between the -592C > A polymorphism and cervical cancer risk [31, 33, 35, 38–40, 42, 44–46]. There were 10 articles that included 1,382 cases and 1,602 controls from Asian populations [31, 34–36, 38, 41–43, 45, 46], 5 articles that included 2,381 cases and 1,396 controls from Caucasian populations [33, 37, 39, 40, 44], and 2 articles that included 274 cases and 251 controls from African populations [27, 32]. Regarding the genotyping method, PCR was used in 13 studies (including ARMS-PCR, PCR-RFLP, PCR Pyrosequencing and Multiplex PCR techniques) [27, 31, 32, 34–38, 42–46], 4 studies reported using the Taqman method [33, 39–41]. The HWE values were calculated based on the genotype distributions of the groups. Some studies deviated from HWE for the IL-10 -1082A > G polymorphism (four studies), -819T > C polymorphism (two studies) and -592C > A polymorphism (one study). A summary of the characteristics of the included studies is shown in Table 1.

**Table 1:** Characteristics of case-control studies on IL-10 -1082A > G, -819T > C and -592C > A polymorphisms and cervical cancer risk

First author	Year	Country	Racial descent	Source of controls	Case	Control	Genotype distribution						*P* for HWE^a^	Genotyping method	NOS
							Case			Control					
							AA	AG	GG	AA	AG	GG			
Stanczuk	2001	Zimbabwe	African	HB	77	69	45	31	1	58	11	0	0.472	ARMS-PCR	6
Roh	2002	Korea	Asian	HB	144	179	144	0	0	179	0	0	NA	PCR-RFLP	4
Govan	2003	South Africa	African	HB	197	182	88	80	29	76	65	41	<0.01	ARMS-PCR	6
Zoodsma	2005	Netherlands	Caucasian	PB	667	606	154	326	187	130	307	169	0.668	Taqman	9
Matsumoto	2010	Japanese	Asian	HB	104	173	73	26	5	156	16	1	0.412	ARMS-PCR	7
Yu	2011	China	Asian	HB	103	115	90	12	1	98	14	3	0.012	ARMS-PCR	6
Wang	2011	China	Asian	PB	186	200	77	85	24	103	76	21	0.222	PCR	7
Barbisan	2012	Argentina	Caucasian	HB	122	176	50	61	11	79	83	14	0.222	PCR Pyrosequencing	6
Singhal	2015	India	Asian	HB	208	250	32	76	100	100	107	43	0.125	PCR-RFLP	7
Zidi	2015	Tunisian	Caucasian	HB	86	126	33	36	17	51	50	25	0.055	TaqMan	6
Torres-Poveda	2015	Mexico	Caucasian	HB	200	200	121	70	9	110	78	12	0.708	TaqMan	8
Zeng	2015	China	Asian	HB	52	50	5	7	40	24	22	0	0.033	TaqMan	5
Bai	2016	China	Asian	HB	165	165	74	75	16	80	72	13	0.563	PCR-RFLP	7
							TT	TC	CC	TT	TC	CC			
Roh	2002	Korea	Asian	HB	144	179	77	56	11	87	77	15	0.724	PCR-RFLP	6
Singh	2009	India.	Asian	HB	150	162	27	67	56	24	61	77	0.046	PCR-RFLP.	6
Singhal	2015	India	Asian	HB	208	250	61	102	45	61	120	69	0.537	PCR-RFLP	7
Zidi	2015	Tunisian	Caucasian	HB	86	126	9	32	45	4	66	56	0.003	TaqMan	5
Torres-Poveda	2016	Mexico	Caucasian	HB	200	200	54	97	49	34	85	81	0.156	TaqMan	8
Bai	2016	China	Asian	HB	165	165	44	75	45	28	73	64	0.362	PCR-RFLP	7
							CC	CA	AA	CC	CA	AA			
Roh	2002	Korea	Asian	HB	144	179	11	56	77	15	77	87	0.724	PCR-RFLP	6
Zoodsma	2005	Netherlands	Caucasian	PB	667	606	393	231	30	405	175	26	0.206	Taqman	9
Ivansson	2007	Sweden	Caucasian	PB	1306	288	736	464	82	162	112	14	0.334	Multiplex PCR	7
Xiong	2010	China	Asian	PB	70	108	12	23	35	13	44	51	0.467	PCR-RFLP	8
Yu	2011	China	Asian	HB	103	115	7	37	59	19	44	52	0.075	ARMS-PCR	7
Shekari	2012	India.	Asian	PB	200	200	16	96	88	17	102	81	0.054	PCR-RFLP	7
Singhal	2015	India	Asian	HB	208	250	85	94	29	60	123	67	0.810	PCR-RFLP	7
Zidi	2015	Tunisian	Caucasian	HB	86	126	45	32	9	57	64	5	0.012	TaqMan	5
Torres-Poveda	2016	Mexico	Caucasian	HB	200	200	44	98	58	85	85	30	0.255	TaqMan	8
Bai	2016	China	Asian	HB	165	165	20	82	63	15	80	70	0.243	PCR-RFLP	7

^a^HWE in control, PB: Population-base control, HB: Hospital-base control and/or healthy base control

### Association between the IL-10 -1082A > G polymorphism and cervical cancer risk

**Figure 2:**
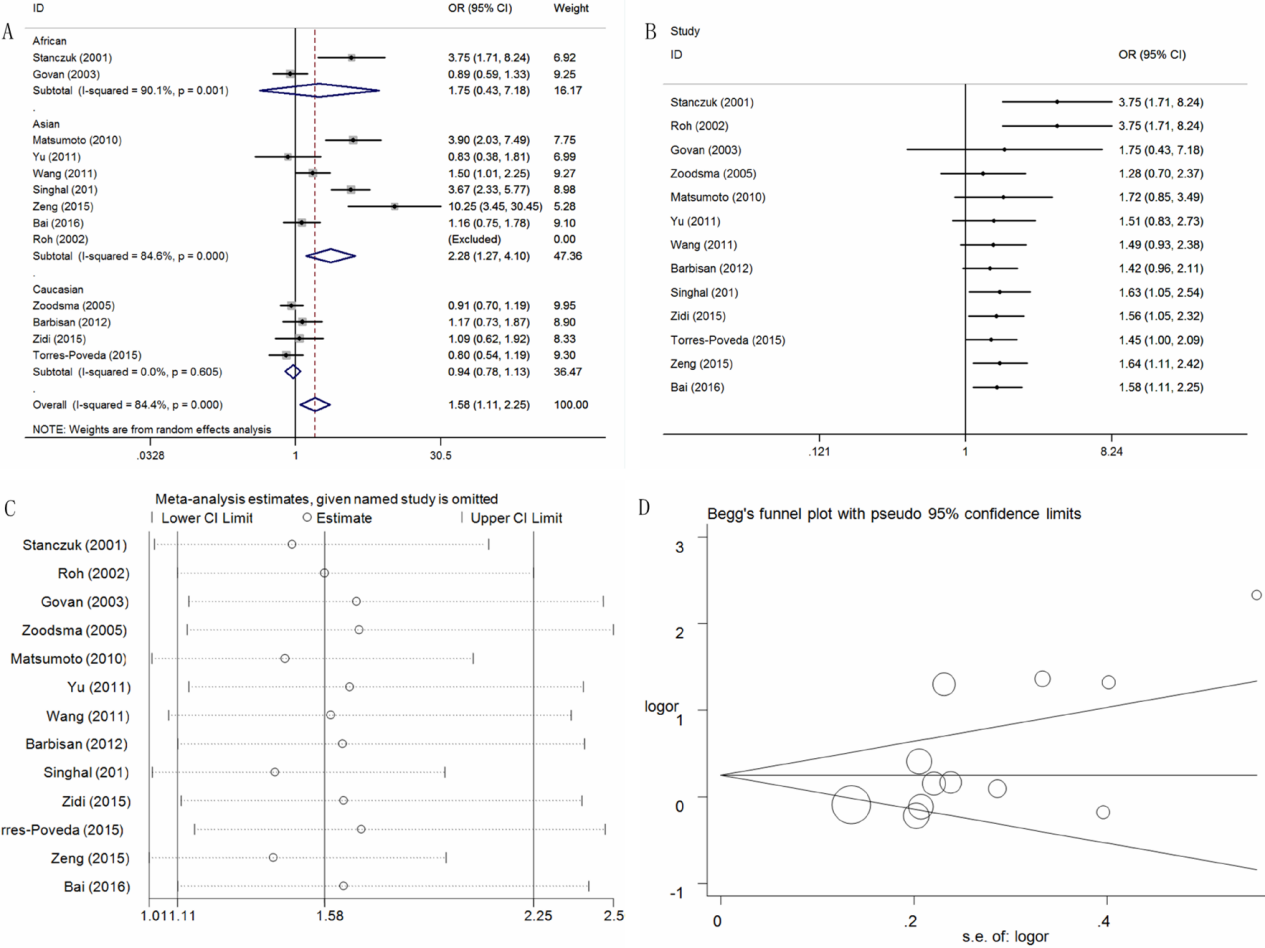
Statistical analysis of the association between the IL-10 -1082A>G polymorphism and cervical cancer risk in the AG+GG vs. AA model (**A**) ORs and 95% CIs; (**B**) cumulative analysis; (**C**) sensitivity analysis; (**D**) publication bias.

A total of thirteen relevant studies, consisting of 2,311 patients and 2,491 controls focused on the association between the IL-10 -1082A > G polymorphism and cervical cancer risk. Overall, a significantly increased risk of cervical cancer was observed in three genetic models (G vs. A: OR = 1.60, 95% CI = 1.12-2.29, *P* = 0.01, I^2^ = 92.3%; AG vs. AA: OR = 1.34, 95%CI = 1.04–1.74, *P* = 0.03, I^2^ = 65.9%; AG+GG vs. AA: OR = 1.58, 95% CI = 1.11–2.25, *P* = 0.02, I^2^ = 84.4%) (Supplementary Table 1, Figure 2A for AG+GG vs. AA model). Subsequent subgroup analyses by ethnicity also revealed a similar risk in Asian populations in all five genetic models (G vs. A: OR = 2.41, 95% CI = 1.26–4.60, *P* = 0.01, I^2^ = 93.3%; AG vs. AA: OR = 1.64, 95% CI = 1.14–2.36, *P* = 0.01, I^2^ = 53.3%; GG vs. AA: OR = 3.75, 95% CI = 1.21–11.61, *P* = 0.02, I^2^ = 85.2%; AG+GG vs. AA: OR = 2.28, 95% CI = 1.27–4.10, *P* = 0.01, I^2^ = 84.6%,; GG vs. AA+AG: OR = 2.94, 95% CI = 1.08-8.03, *P* = 0.04, I^2^ = 83.7%). Moreover, a significant risk of cervical cancer was also seen in all five genetic models for the HWE, the hospital control and the PCR genotyping groups (Supplementary Table 1). Heterogeneity was observed in all five genetic models. Meta-regression analyses were conducted, but the results failed to identify any factors contributing to the observed heterogeneity.

A cumulative analysis by publication date demonstrated that cancer risk increased gradually and became positive following the study conducted by Singhal et al. in 2012 were included (Figure 2B for AG+GG vs. AA model). A sensitivity analysis revealed that no single study qualitatively changed the pooled ORs, indicating that the results of this meta-analysis were stable (Figure 2C for AG+GG vs. AA model).

Publication bias was assessed with Funnel plots. Plot asymmetry was observed only in the AG+GG vs. AA model, which was redressed in the subgroup analysis by ethnicity (Figure 2D for AG+GG vs. AA model). These results were further supported by Egger’s tests (G vs. A: *P* = 0.12; AG vs. AA: *P* = 0.06; GG vs. AA: *P* = 0.34; AG+GG vs. AA: *P* = 0.03; GG vs. AA+AG: *P* = 0.46).

### Association between the IL-10 -819T > C polymorphism and cervical cancer risk

**Figure 3:**
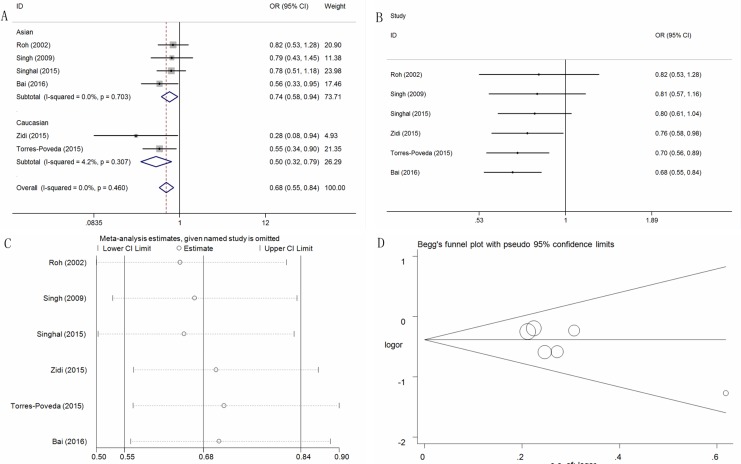
Statistical analysis of the association between the IL-10 -819T>C polymorphism and cervical cancer risk in the TC+CC vs. TT model (**A**) ORs and 95% CIs; (**B**) cumulative analysis; (**C**) sensitivity analysis; (**D**) publication bias.

Six studies involving 953 cases and 1,082 controls were included in this meta-analysis to assess the association between the IL-10 -819T > C polymorphism and cervical cancer risk. Interestingly, all genetic models indicated that they confer a protective effect against cervical cancer occurrence (C vs. T, OR = 0.74, 95% CI = 0.65-0.84, *P* < 0.01, I^2^ = 23.8%; TC vs. TT, OR = 0.76, 95%CI = 0.61-0.95, P = 0.02, I^2^ = 2.6%; CC vs. TT, OR = 0.53, 95% CI = 0.41-0.70, *P* < 0.01, I^2^ = 0%; TC+CC vs. TT, OR = 0.68, 95%CI = 0.55-0.84, *P* < 0.01, I^2^ = 0%; CC vs. TT+TC, OR = 0.71, 95%CI = 0.54–0.95, P = 0.02, I^2^ = 48.7%) (Supplementary Table 1, Figure 3A for TC+CC vs. TT model). Furthermore, subgroup analyses based on HWE status, the ethnicity, the control design and the genotyping method all indicated the same protective effect (Supplementary Table 1).

The cumulative analysis also indicated some change only in the recessive model (Figure 3B for TC+CC vs. TT model). A sensitivity analysis was conducted, and no conspicuous change in the pooled ORs was detected except in the recessive model (Figure 3C for TC+CC vs. TT model). Moreover, no publication bias was observed, indicating that the results were statistically robust (C vs. T: *P* = 0.23; TC vs. TT: *P* = 0.06; CC vs. TT: *P* = 0.96; TC+CC vs. TT: *P* = 0.11; CC vs. TT+TC: *P* = 0.26) (Figure 3D for TC+CC vs. TT model).

### Association between the IL-10 -592C > A polymorphism and cervical cancer risk

**Figure 4:**
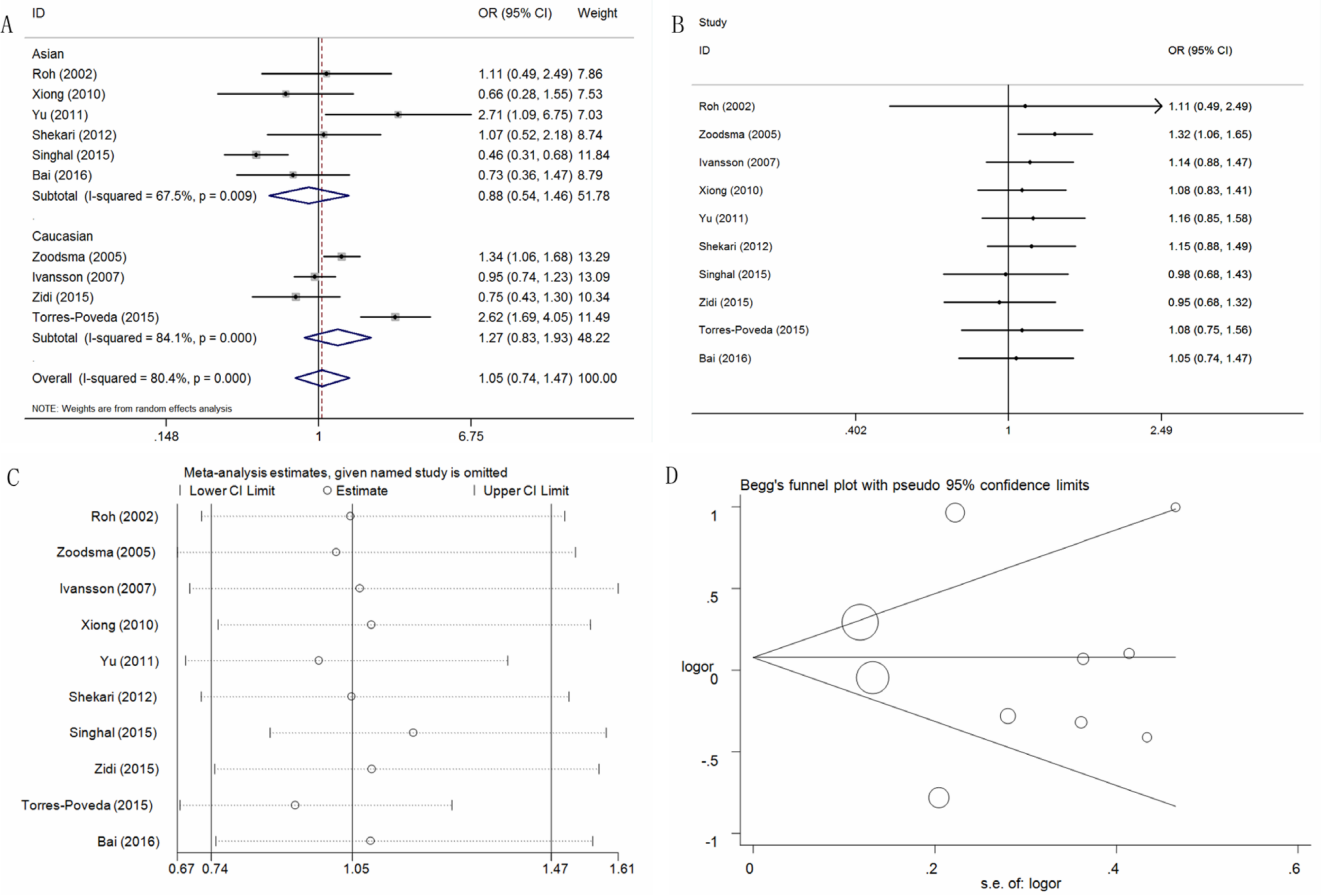
Statistical analysis of the association between the IL-10 -592C>A polymorphism and cervical cancer risk in the CA+AA vs. CC model (**A**) ORs and 95% CIs; (**B**) cumulative analysis; (**C**) sensitivity analysis; (**D**) publication bias.

For the IL-10 -592C > A polymorphism, ten studies consisting of 3,149 cases and 2,237 controls were pooled in the meta-analysis to assess whether this IL-10 -592C > A polymorphism was associated with cervical cancer risk. Overall, no significant association was observed in any of the five models (Supplementary Table 1; Figure 4A for CA+AA vs. CC model). Only two genetic models (for AA vs. CC, OR = 1.86, 95% CI = 1.02-3.39, *P* = 0.04, I^2^ = 69.6%; for AA vs. CC+CA, OR = 1.62, 95%CI = 1.06-2.49, *P* = 0.03, I^2^ = 46.6%) revealed an increased risk for cervical cancer in the Caucasian population group. Additional subgroup analyses were conducted based on HWE status, the control design and the genotyping method, but no significant associations were observed. The pooled ORs did not exhibit any changes following sensitivity or cumulative analyses, and no publication bias was observed (Figure 4B and 4C for CA+AA vs. CC model). Moreover, no publication bias was observed (A vs. C: P = 0.96; CA vs. CC: P = 0.63; AA vs. CC: *P* = 0.58; CA+AA vs. CC: *P* = 0.82; AA vs. CC+CA: *P* = 0.34) (Figure 4D for CA+AA vs. CC model).

## DISCUSSION

Genetic factors are now known to be important variables affecting the susceptibility of patients to various diseases and have been paid an increasing amount of attention [47, 48]. Inflammatory and immune cytokines play important roles during the process of tumorigenesis transition from normal epithelium to malignant tumors, and these cytokines can promote this process by inducing angiogenesis, compensatory cell proliferation, DNA damage, or the accumulation of gene mutations [49]. Gene mutations, especially polymorphisms in the promoter region, can affect the process of gene transcription, resulting in abnormal expression of the corresponding mRNAs and dysfunction of the expressed proteins. These mutations can also influence the susceptibility of individuals to cancer [50, 51].

Since 2001, a large number of molecular epidemiological case-control studies have been conducted to explore the association between IL-10 polymorphisms and cervical cancer risk, but the results have been inconsistent. In terms of the IL-10 -1082A > G polymorphism, Singhal et al. reported a case-control study in an Indian population, the AG and GG genotypes may significantly increase the risk of cervical cancer development compared with the AA genotype (AG vs. AA: OR = 2.2, 95%CI = 1.35–3.64; GG vs. AA: OR = 7.26, 95% CI = 4.2-12.4) [38]. A similarly increased risk was also observed in other studies. In contrast, other studies reported a negative association between the IL-10–1082A > G polymorphism and cervical cancer risk. For -819T > C and -592C > A polymorphisms, there is still controversy regarding the relationship between these two polymorphisms and cervical cancer susceptibility.

In 2013, Ni et al. published the first meta-analysis examining the association between IL-10 gene polymorphisms and cervical cancer risk. Their meta-analysis included eight studies comprising 1,498 cases and 1,608 controls for the IL-10 -1082A > G polymorphism, as well as five studies involving 2,396 cases and 1,388 controls for the IL-10 -592C > A polymorphism [28]. The researchers found that the IL-10 -592C > A polymorphism was associated with an increased risk of cervical cancer development. No significant association was found for the IL-10 -1082A > G polymorphism [28]. In 2014, Zhang et al. conducted another meta-analysis to evaluate the association between the IL-10 -1082A > G polymorphism and cervical cancer risk. This study included eight case-control studies comprising 1,983 cases and 1,618 controls; they also reported no association between this polymorphism and cervical cancer susceptibility [29]. Moreover, the association between the IL-10–819T > C polymorphism and cervical cancer risk was also explored in the subgroup analysis by Yu et al. in 2013. This analysis only included three case-control studies, with 1,895 subjects, and indicated that there was a positive association with cervical cancer in some genetic models [52].

Compared with the previous meta-analyses, our meta-analysis relied on a more scientifically sound retrieval strategy and included more research studies (seventeen publications involving 7,286 individuals) to accurately assess the associations between the IL-10 -1082A > G, -819T > C and -592C > A polymorphisms and cervical cancer risk. Our results suggest that the IL-10–1082A > G polymorphism may be associated with an increased risk of cervical cancer development. In contrast, the IL-10–819T > C polymorphism may have a protective effect against cervical cancer development, especially in Asian populations. In the stratified analysis, the heterogeneity between the included IL-10–1082A > G polymorphism studies was moderately, and successfully reduced by the subgroup analysis based on ethnicity. This suggests that ethnic diversity maybe a potential heterogeneity factor. Moreover, after conducting the sensitivity and publication bias analyses, no significant alterations or bias were observed for any of the three polymorphisms, presenting the stability of the results of our meta-analysis.

To our knowledge, this is the first meta-analysis to examine the association between IL-10 polymorphisms and cervical cancer risk, including the three most common polymorphic loci (IL-10 -1082A > G, -819T > C and -592C > A). However, there are some limitations of this study that should be addressed. First, only seventeen publications were included in our meta-analysis. The limited number of studies and sample size for each polymorphic locus may reduce the reliability of the results and affect the assessment of associations between these IL-10 polymorphisms and cervical cancer susceptibility. Second, additional risk factors, such as smoking, drinking, and HPV infection, were not considered. The interactions between genetic and environmental factors and cancer development could not be evaluated in our analysis. Third, the association between the IL-10 -1082A > G, -819T > C and -592C > A polymorphisms and cervical cancer risk were analyzed separately, and the influence of the haplotype and gene-gene interactions was not analyzed due to an insufficient amount of data. Fourth, the heterogeneity that exists between the IL-10–1082A > G polymorphism studies could influence the current results and distort the conclusions. In this meta-regression analysis, we could not find the source of the heterogeneity, although it could be reduced in the subgroup analysis.

In summary, our meta-analysis suggests that the IL-10–1082A > G and -819T > C polymorphisms are associated with cervical cancer susceptibility, but with contradictory effects. In contrast, no significant association was found between the IL-10–592C > A polymorphism and cervical cancer susceptibility.

## MATERIALS AND METHODS

### Literature search strategy

A comprehensive literature search (up until June 1^st^, 2017) was independently performed by two of the authors, without restrictions on the geographic region or language of publications from the following online databases: PubMed, Embase, Science Citation Index (SCI), CNKI and Wanfang. References appearing in relevant reports and recent reviews were all screened to identify potential articles of interest. The search terms“Interleukin-10”, “polymorphism” AND “cervical cancer”, and the following search strategy were used (in PubMed, for example): #1 Interleukin-10, #2 IL-10, #3 rs1800870, #4 rs1800871, #5 rs1800872, #6 #1 OR #2 OR #3 OR #4 OR #5, #7 polymorphism, #8 variant, #9 mutation, #10 #7 OR #8 OR #9, #11 cervical cancer, #12 cervical tumor, #13 cervical neoplasm, #14 #11 OR #12 OR #13, #15 #6 AND #10 AND #14.

### Eligibility criteria

To be included in this study, publications had to meet the following inclusion criteria: 1) the study design had to be a case-control study (including retrospective or prospective studies); 2) the focus of the study had to be on IL-10 promoter polymorphisms (-1082A > G, -819T > C and -592C > A); 3) the case group had to include women with cervical cancer and the control group had to consist of women without cervical cancer; 4) the reports had to include sufficient information on the frequency distribution of different genotypes in order to calculate the odds ratios (ORs) and 95% confidence intervals (CIs); and 5) in the case that there were duplicate studies, we included the most recent or those with the largest samples sizes.

### Data extraction and quality evaluation

Two investigators (Guo and Wen), independently extracted the following information from all of the included studies: first author, year of publication, study country or region where the study was performed, ethnicity (Asian, Caucasian or African), the source of the controls, the sample sizes of patients and controls, data on the frequency distribution of different genotypes, the Hardy-Weinberg equilibrium (HWE) for the controls, and the genotyping method. In addition, the modified Newcastle-Ottawa scale (NOS) was employed by the first two authors in order to evaluate the quality of the included studies [53]. The scores ranged from 0 points (worst) to 10 points (best). Studies with a score of 7 or higher were classified as high quality (Table 2).

**Table 2:** Scale for quality evaluation

Criteria	Score
**Representativeness of cases** Consecutive/randomly selected cases with clearly defined sampling frame Not consecutive/randomly selected case or without clearly defined sampling frame Not described	2 1 0
**Source of controls** Population-base control Hospital-base control and/or healthy base control Not described	2 1 0
**Hardy-Weinberg equilibrium in controls** Hardy-Weinberg equilibrium Hardy-Weinberg disequilibrium Not available	2 1 0
**Genotyping examination** Genotyping done under “blinded” condition and repeated again Genotyping done under “blinded” condition or repeated again Unblinded done or not mentioned and unrepeated	2 1 0
**Association assessment** Assess association between genotypes and cancer with appropriate statistics and adjustment for confounders Assess association between genotypes and cancer with appropriate statistics and without adjustment for confounders Inappropriate statistics used	2 1 0

### Statistical analysis

ORs with 95% CIs were calculated to assess the strength of the association between the IL-10 -1082A > G, -819T > C and -592C > A polymorphisms and cervical cancer susceptibility. For the IL-10 -1082A > G polymorphism, the five following genetic models were used: allele contrast model (G vs. A), co-dominant models (AG vs. AA and GG vs. AA), dominant model (AG+GG vs. AA), and recessive model (GG vs. AA+AG). The same genetic models were also used to assess the IL-10 -819T > C and -592C > A polymorphisms. Subgroup analyses were performed according to HWE status, ethnicity difference, control designs, and genotyping methods. Heterogeneity between studies was determined via a Cochran’s *Q* test and the I^2^ statistic [54]. A fixed-effect model (the Mantel-Haenszel method) was used when the *P*-value was more than 0.10 and the I^2^ was less than 40% [55]. Otherwise, a random-effects model (the DerSimonian and Laird method) was adopted [56]. Meta-regression analyses were conducted to explore the potential factors that contribute to heterogeneity. Furthermore, cumulative meta-analyses were conducted to observe whether the trend changed with the addition of studies. Sensitivity analyses were also conducted to evaluate the robustness of our results. Egger’s linear regression and Begg’s funnel plots were used to examine any potential publication bias [57, 58]. All statistical calculations were performed with STATA version 14.0 (Stata Corporation, College Station, TX, USA). A two-sided *P* value < 0.05 was considered significant.

## SUPPLEMENTARY MATERIALS TABLE




