# Supplementary material for: Significant association between interleukin-10 gene polymorphisms and cervical cancer risk: a meta-analysis
Source: Oncotarget. 2018 Jan 12;9(15):12365–75. doi: 10.18632/oncotarget.24193 (PMC5844753; doi:10.18632/oncotarget.24193)
Supplement: Supplementary file 1 [file oncotarget-09-12365-s001.pdf]

# Significant association between interleukin-10 gene polymorphisms and cervical cancer risk: a meta-analysis

## SUPPLEMENTARY MATERIALS

**Supplementary Table 1: Summary ORs and 95% CI of IL-10 -1082A>G, -819 T > C and -592 C > A polymorphisms and cervical cancer risk.** See Supplementary Table 1
