# Supplementary material for: Significant association between interleukin-10 gene polymorphisms and cervical cancer risk: a meta-analysis
Source: Oncotarget. 2018 Jan 12;9(15):12365–75. doi: 10.18632/oncotarget.24193 (PMC5844753; doi:10.18632/oncotarget.24193)
Supplement: Supplementary file 2 [file oncotarget-09-12365-s002.docx]

**Supplementary Table 1: Summary ORs and 95% CI of IL-10 -1082A>G, -819 T>C and -592 C>A polymorphisms and cervical cancer risk**

|  | N* | G vs. A | | | |  | AG vs. AA | | | |  | GG vs. AA | | | |  | AG+GG vs. AA | | | |  | GG vs. AA+AG | | | | |
| --- | --- | --- | --- | --- | --- | --- | --- | --- | --- | --- | --- | --- | --- | --- | --- | --- | --- | --- | --- | --- | --- | --- | --- | --- | --- | --- |
| -1082A>G |  | OR | 95% CI | *P* | *I^2^* |  | OR | 95% CI | *P* | *I^2^* |  | OR | 95% CI | *P* | *I^2^* |  | OR | 95% CI | *P* | *I^2^* |  | OR | | 95% CI | *P* | *I^2^* |
| Total | 13 | 1.60 | 1.12-2.29 | 0.01 | 92.3 |  | 1.34 | 1.04-1.74 | 0.03 | 65.9 |  | 1.68 | 0.91-3.11 | 0.10 | 85.1 |  | 1.58 | 1.11-2.25 | 0.01 | 84.4 |  | 1.46 | | 0.86-.2.48 | 0.16 | 83.5 |
| HWE-yes | 9 | 1.49 | 1.06-2.10 | 0.02 | 90.2 |  | 1.43 | 1.04-1.96 | 0.03 | 74.5 |  | 1.68 | 0.89-3.16 | 0.11 | 83.9 |  | 1.57 | 1.07-2.30 | 0.02 | 84.8 |  | 1.47 | | 0.88-2.46 | 0.15 | 80.7 |
| HWE-no | 4 | 2.12 | 0.35-12.73 | 0.41 | 96.8 |  | 1.07 | 0.73-1.56 | 0.74 | 0 |  | 3.56 | 0.08-149.91 | 0.51 | 91.5 |  | 1.80 | 0.51-6.31 | 0.36 | 88.8 |  | 3.45 | | 0.07-179.78 | 0.54 | 92.6 |
| Ethnicity |  |  |  |  |  |  |  |  |  |  |  |  |  |  |  |  |  |  |  |  |  |  | |  |  |  |
| African | 2 | 1.52 | 0.39-5.84 | 0.54 | 91.6 |  | 1.88 | 0.56-6.25 | 0.30 | 85.8 |  | 0.66 | 0.38-1.14 | 0.13 | 18.1 |  | 1.75 | 0.43-7.18 | 0.44 | 90.1 |  | 0.62 | | 0.37-1.04 | 0.07 | 0 |
| Asian | 7 | 2.41 | 1.26-4.60 | 0.01 | 93.3 |  | 1.64 | 1.14-2.36 | 0.01 | 53.3 |  | 3.75 | 1.21-11.61 | 0.02 | 85.2 |  | 2.28 | 1.27-4.10 | 0.01 | 84.6 |  | 2.94 | | 1.08-8.03 | 0.04 | 83.7 |
| Caucasian | 4 | 0.97 | 0.86-1.10 | 0.66 | 0 |  | 0.94 | 0.77-1.14 | 0.52 | 0 |  | 0.95 | 0.73-1.23 | 0.68 | 0 |  | 0.94 | 0.78-1.13 | 0.52 | 0 |  | 1.00 | | 0.80-1.24 | 0.98 | 0 |
| Design |  |  |  |  |  |  |  |  |  |  |  |  |  |  |  |  |  |  |  |  |  |  | |  |  |  |
| HB | 11 | 1.76 | 1.09-2.84 | 0.02 | 93.0 |  | 1.42 | 1.04-1.94 | 0.03 | 65.6 |  | 2.01 | 0.85-4.74 | 0.11 | 86.4 |  | 1.72 | 1.10-2.70 | 0.02 | 85.5 |  | 1.72 | | 0.80-3.68 | 0.14 | 85.1 |
| PB | 2 | 1.11 | 0.82-1.50 | 0.52 | 69.7 |  | 1.13 | 0.68-1.86 | 0.64 | 74.0 |  | 1.09 | 0.70-1.72 | 0.70 | 43.4 |  | 1.14 | 0.70-1.86 | 0.60 | 76.4 |  | 1.04 | | 0.83-1.30 | 0.75 | 0 |
| Genotyping method |  |  |  |  |  |  |  |  |  |  |  |  |  |  |  |  |  |  |  |  |  |  | |  |  |  |
| PCR | 9 | 1.55 | 1.01-2.38 | 0.04 | 89.9 |  | 1.58 | 1.15-2.18 | 0.01 | 63.5 |  | 1.77 | 0.76-4.13 | 0.18 | 84.9 |  | 1.70 | 1.11-2.60 | 0.02 | 82.1 |  | 1.49 | | 0.71-3.12 | 0.29 | 83.1 |
| Taqman | 4 | 1.76 | 0.83-3.73 | 0.14 | 95.1 |  | 0.91 | 0.74-1.13 | 0.39 | 0 |  | 1.52 | 0.55-4.22 | 0.43 | 83.6 |  | 1.35 | 0.73-2.48 | 0.34 | 84.6 |  | 1.49 | | 0.58-3.83 | 0.41 | 83.7 |
| NOS evaluation |  |  |  |  |  |  |  |  |  |  |  |  |  |  |  |  |  |  |  |  |  |  | |  |  |  |
| NOS ≥7 | 6 | 1.52 | 0.96-2.39 | 0.07 | 93.3 |  | 1.38 | 0.94-2.03 | 0.10 | 79.0 |  | 1.88 | 0.81-4.40 | 0.14 | 89.7 |  | 1.57 | 0.95-2.57 | 0.08 | 88.8 |  | 1.62 | | 0.82-3.22 | 0.16 | 87.6 |
| NOS < 7 | 7 | 1.75 | 0.87-3.52 | 0.12 | 92.8 |  | 1.30 | 0.92-1.84 | 0.14 | 39.8 |  | 1.55 | 0.54-4.45 | 0.42 | 78.4 |  | 1.62 | 0.51-2.88 | 0.10 | 80.8 |  | 1.44 | | 0.51-4.10 | 0.49  49 | 80.4 |
|  |  |  |  |  |  |  |  |  |  |  |  |  |  |  |  |  |  |  |  |  |  |  | |  |  |  |
| -819 T>C |  | C vs. T | | | |  | TC vs. TT | | | |  | CC vs. TT | | | |  | TC+CC vs.TT | | | |  | CC vs. TT+TC | | | | |
| Total | 6 | 0.74 | 0.65-0.84 | <0.01 | 23.8 |  | 0.76 | 0.61-0.95 | 0.02 | 2.6 |  | 0.53 | 0.41-0.70 | <0.01 | 0 |  | 0.68 | 0.55-0.84 | <0.01 | 0 |  | 0.71 | 0.54-0.95 | | 0.02 | 48.7 |
| HWE-yes | 4 | 0.71 | 0.62-0.83 | <0.01 | 29.1 |  | 0.77 | 0.60-0.99 | 0.04 | 0 |  | 0.53 | 0.39-0.71 | <0.01 | 10.8 |  | 0.69 | 0.55-0.87 | <0.01 | 0 |  | 0.61 | 0.48-0.78 | | <0.01 | 0 |
| HWE-no | 2 | 0.84 | 0.65-1.09 | 0.18 | 17.7 |  | 0.51 | 0.12-2.20 | 0.36 | 77.3 |  | 0.57 | 0.32-1.00 | 0.05 | 0 |  | 0.54 | 0.20-1.44 | 0.22 | 55.9 |  | 0.93 | 0.45-1.92 | | 0.85 | 75.6 |
| Ethnicity |  |  |  |  |  |  |  |  |  |  |  |  |  |  |  |  |  |  |  |  |  |  |  | |  |  |
| Asian | 4 | 0.76 | 0.66-0.89 | <0.01 | 0 |  | 0.82 | 0.63-1.05 | 0.12 | 0 |  | 0.61 | 0.45-0.83 | <0.01 | 0 |  | 0.74 | 0.58-0.94 | 0.01 | 0.0 |  | 0.68 | 0.53-0.87 | | 0.02 | 0 |
| Caucasian | 2 | 0.75 | 0.44-1.28 | 0.30 | 77.0 |  | 0.45 | 0.14-1.43 | 0.18 | 67.1 |  | 0.38 | 0.23-0.63 | <0.01 | 0 |  | 0.50 | 0.32-0.79 | <0.01 | 4.02 |  | 0.80 | 0.28-2.24 | | 0.67 | 88.7 |
| Genotyping method |  |  |  |  |  |  |  |  |  |  |  |  |  |  |  |  |  |  |  |  |  |  |  | |  |  |
| PCR | 4 | 0.76 | 0.66-0.89 | <0.01 | 0 |  | 0.82 | 0.63-1.05 | 0.12 | 0 |  | 0.61 | 0.45-0.83 | <0.01 | 0 |  | 0.74 | 0.58-0.94 | 0.01 | 0.0 |  | 0.68 | 0.53-0.87 | | 0.02 | 0 |
| Taqman | 2 | 0.75 | 0.44-1.28 | 0.30 | 77.0 |  | 0.45 | 0.14-1.43 | 0.18 | 67.1 |  | 0.38 | 0.23-0.63 | <0.01 | 0 |  | 0.50 | 0.32-0.79 | <0.01 | 4.02 |  | 0.80 | 0.28-2.24 | | 0.67 | 88.7 |
| NOS evaluation |  |  |  |  |  |  |  |  |  |  |  |  |  |  |  |  |  |  |  |  |  |  |  | |  |  |
| NOS ≥7 | 3 | 0.68 | 0.58-0.80 | <0.01 | 24.9 |  | 0.75 | 0.56-1.01 | 0.06 | 0.0 |  | 0.49 | 0.36-0.68 | <0.01 | 2.4 |  | 0.64 | 0.49-0.84 | <0.01 | 0.0 |  | 0.59 | 0.46-0.76 | | <0.01 | 0 |
| NOS < 7 | 3 | 0.85 | 0.69-1.05 | 0.12 | 0 |  | 0.69 | 0.37-1.29 | 0.25 | 56.2 |  | 0.64 | 0.40-1.02 | 0.06 | 0 |  | 0.74 | 0.53-1.04 | 0.08 | 26.8 |  | 0.92 | 0.57-1.49 | | 0.73 | 51.2 |
|  |  |  |  |  |  |  |  |  |  |  |  |  |  |  |  |  |  |  |  |  |  |  |  | |  |  |
| -592 C>A |  | A vs. C | | | |  | CA vs. CC | | | |  | AA vs. CC | | | |  | CA+AA vs. CC | | | |  | AA vs. CC+CA | | | | |
| Total | 10 | 1.09 | 0.86-1.37 | 0.49 | 83.3 |  | 0.99 | 0.73-1.35 | 0.97 | 72.5 |  | 1.21 | 0.72-2.03 | 0.47 | 81.0 |  | 1.05 | 0.74-1.74 | 0.79 | 80.4 |  | 1.18 | 0.87-1.59 | | 0.28 | 69.1 |
| HWE-yes | 9 | 1.10 | 0.85-1.41 | 0.47 | 85.1 |  | 1.05 | 0.76-1.45 | 0.78 | 73.0 |  | 1.15 | 0.66-1.98 | 0.62 | 82.6 |  | 1.09 | 0.75-1.57 | 0.66 | 81.9 |  | 1.13 | 0.84-1.52 | | 0.43 | 69.9 |
| HWE-no | 1 | 0.99 | 0.64-1.51 | 0.95 | NA |  | 0.63 | 0.36-1.13 | 0.12 | NA |  | 2.28 | 0.71-7.28 | 0.16 | NA |  | 0.75 | 0.43-1.30 | 0.31 | NA |  | 2.83 | 0.91-8.76 | | 0.07 | NA |
| Ethnicity |  |  |  |  |  |  |  |  |  |  |  |  |  |  |  |  |  |  |  |  |  |  |  | |  |  |
| Asian | 6 | 0.97 | 0.71-1.34 | 0.87 | 80.9 |  | 0.83 | 0.56-1.23 | 0.35 | 42.6 |  | 0.89 | 0.46-1.71 | 0.73 | 76.8 |  | 0.88 | 0.54-1.46 | 0.63 | 67.5 |  | 0.99 | 0.70-1.40 | | 0.93 | 69.0 |
| Caucasian | 4 | 1.27 | 0.94-1.71 | 0.13 | 81.5 |  | 1.17 | 0.77-1.77 | 0.46 | 81.8 |  | 1.86 | 1.02-3.39 | 0.04 | 69.6 |  | 1.27 | 0.83-1.93 | 0.27 | 84.1 |  | 1.62 | 1.06-2.49 | | 0.03 | 46.6 |
| Design |  |  |  |  |  |  |  |  |  |  |  |  |  |  |  |  |  |  |  |  |  |  |  | |  |  |
| HB | 6 | 1.10 | 0.71-1.69 | 0.68 | 90.2 |  | 1.02 | 0.58-1.79 | 0.95 | 80.0 |  | 1.33 | 0.52-3.39 | 0.55 | 89.2 |  | 1.09 | 0.56-2.14 | 0.80 | 87.5 |  | 1.22 | 0.73-2.05 | | 0.45 | 82.6 |
| PB | 4 | 1.11 | 0.98-1.25 | 0.10 | 0 |  | 1.03 | 0.75-1.42 | 0.84 | 56.5 |  | 1.14 | 0.82-1.58 | 0.44 | 0 |  | 1.08 | 0.83-1.40 | 0.55 | 43.1 |  | 1.16 | 0.90-1.50 | | 0.25 | 0 |
| Genotyping method |  |  |  |  |  |  |  |  |  |  |  |  |  |  |  |  |  |  |  |  |  |  |  | |  |  |
| PCR | 7 | 0.98 | 0.76-1.25 | 0.85 | 77.7 |  | 0.83 | 0.69-1.01 | 0.06 | 38.0 |  | 0.94 | 0.54-1.64 | 0.83 | 75.3 |  | 0.88 | 0.60-1.28 | 0.51 | 65.7 |  | 1.02 | 0.75-1.40 | | 0.89 | 64.9 |
| Taqman | 3 | 1.38 | 0.94-2.02 | 0.10 | 81.0 |  | 1.28 | 0.73-2.25 | 0.40 | 81.9 |  | 2.14 | 0.94-4.85 | 0.07 | 75.4 |  | 1.41 | 0.79-2.51 | 0.25 | 84.6 |  | 1.78 | 0.97-3.26 | | 0.06 | 60.8 |
| NOS evaluation |  |  |  |  |  |  |  |  |  |  |  |  |  |  |  |  |  |  |  |  |  |  |  | |  |  |
| NOS ≥7 | 8 | 1.09 | 0.83-1.44 | 0.54 | 86.9 |  | 1.05 | 0.74-1.49 | 0.77 | 76.3 |  | 1.14 | 0.62-2.09 | 0.67 | 84.8 |  | 1.09 | 0.73-1.62 | 0.68 | 84.1 |  | 1.12 | 0.79-1.58 | | 0.53 | 73.5 |
| NOS < 7 | 2 | 1.08 | 0.83-1.41 | 0.57 | 0 |  | 0.73 | 0.45-1.18 | 0.20 | 0 |  | 1.50 | 0.76-2.96 | 0.24 | 0 |  | 0.85 | 0.54-1.34 | 0.48 | 0 |  | 1.57 | 0.73-3.36 | | 0.25 | 46.4 |

^*^ Numbers of comparisons
